# Reminders of Mortality Alter Pain-Evoked Potentials in a Chinese Sample

**DOI:** 10.3389/fpsyg.2018.01667

**Published:** 2018-09-07

**Authors:** Chenbo Wang, Jing Tian

**Affiliations:** ^1^Faculty of Education, East China Normal University, Shanghai, China; ^2^Key Laboratory of Brain Functional Genomics (MOE&STCSM), Shanghai Changning-ECNU Mental Health Center, School of Psychology and Cognitive Science, East China Normal University, Shanghai, China; ^3^Teachers College, Columbia University, New York, NY, United States

**Keywords:** pain, mortality salience, ERP, priming, culture

## Abstract

Pain is of evolutionary importance to human survival. However, the perception of pain could be changed when death-related thoughts are accessible. Although the influence of mortality salience (MS) on pain processing has been investigated in Westerners recently, it is unclear whether this effect is constrained by specific culture context since humans may employ cultural worldviews to defend the existence problem. The current study tested whether and how MS affected pain processing in a Chinese male sample. We primed participants with sentences indicating MS or negative affect (NA) on either of two days. Both before and after the priming, event-related potentials (ERPs) elicited by painful and non-painful electrical stimulations were recorded. Results showed that pain-evoked potentials were identified as an early negative complex N60-P90-N130 and a late positivity P260. Pain-evoked N130 after MS priming was larger than that after NA priming. Meanwhile, pain-evoked P260 decreased after MS priming but not after NA priming. These findings indicate that reminders of mortality affect both early sensory and late cognitive neural responses related to physical pain. Although previous studies reporting an increased effect of MS on perceived pain intensity in Westerners, we found an unchanged or possibly reduced effect in Chinese. Thus, the current work provides insight into a culture-sensitive perspective on how pain processing would be modulated when existential problem occurs.

## Introduction

Pain perception and its underlying neural activities can be affected by multiple cognitive factors (Gatchel et al., 2007; Williams and Craig, 2016), including attention (Kakigi et al., 2005), memory (Eich et al., 1985), appraisal and beliefs (Wager et al., 2004). For example, attention to or distraction from a target stimulus influences the intensity of painful feelings (Kakigi et al., 2005), as well as pain-evoked brain potentials (Eimer and rster, 2003). Pain perception is also found to be modulated by intended self-regulation and unintended anticipation (e.g., Wager et al., 2004; Woo, et al., 2015; Zhao et al., 2017). Such cognitive modulations are thought to be related to brain activities in several areas, especially in the prefrontal cortex (Woo, et al., 2015).

The cognitive modulations of pain processing may be associated with the meaning ascribed to pain by an individual (Sharp, 2001). By alerting us to actual or possible tissue damage, pain provides important meaning for the existence of humans and other animals (Cassel, 1982). Meanwhile, reconceptualising pain as a challenge rather than a threat could change the painful feeling in a positive direction (Leknes and Bastian, 2014). For example, participants who explicitly stated the challenge of a cold-pressor test in which they were asked to keep their hand in ice-cold water as along as possible felt better after undergoing the pain challenge (Franklin et al., 2010). Likewise, in situations where people are confronted with existential threat, they may re-appraise the meaning of pain. This raises an important question that how mortality salience (MS) would affect pain processing.

Recent social neuroscience research has tackled the neural correlates of death-related thought and its impact on social/affective processes (Han et al., 2010; Henry et al., 2010; Quirin et al., 2011; Klackl et al., 2013; Luo et al., 2014; Valentini et al., 2014). Valentini et al. (2014, 2015, 2017) conducted a series of studies to explore the influence of MS on pain processing. They found that reminders of mortality increased pain intensity rating and enhanced theta oscillatory activity, slow wave negativity and frontal delta band activity responding to nociceptive stimulation (Valentini et al., 2014, 2015). The slow wave and the delta spectral activity were, respectively, associated with increased state anxiety and higher self-esteem. As posited by the terror management theory (TMT), humans employ proximal defenses (distraction or rationalization) and distal defenses (self-esteem or cultural worldview) to keep death-related awareness under control (Pyszczynski et al., 1999; Greenberg et al., 2000). Therefore, these results support that MS increases painful feelings by employing both proximal and distal defenses.

Meanwhile, it is intriguing to test an alternative possibility that MS may reduce painful feelings under certain circumstance. According to the TMT, human beings employ cultural worldviews to defend the existence problem (Greenberg et al., 2000). When confronted with death-related thoughts, people in different regions may employ different cultural worldviews. The long-term/short-term orientation or Confucian dynamism, a cultural dimension Hofstede proposed (Hofstede, 2001), claims that East Asia culture encourages delayed gratification of material and mental achievements among its members. On the other hand, painful rituals are also prevalent within certain religious traditions, such as Buddhism in China. Tolerance of pain is linked to a perception that a person is noble and heroic (Morris, 1991). Thus, we hypothesize that when Chinese participants are reminded of death, they may re-appraise the meaning of pain (such as being recognized as a challenge instead of a threat), and therefore decrease the ratings of pain intensity.

In the current study, we aimed at investigating whether and how MS influences pain processing in a Chinese sample. MS was induced by asking participants to read death-related statements (Luo et al., 2014). To measure the neural processing of pain, brain potentials evoked by painful electrical shocks were tracked by electroencephalograph (EEG). When electrical painful compared to non-painful stimuli were delivered to the hand, event-related potentials (ERPs) evoked by pain were identified in previous studies, e.g., the N60, N120, and P170 (Babiloni et al., 2001); the N60, P90, N130, and P260/P300 (Zohsel et al., 2008; Wang et al., 2014). The early neural responses to painful stimuli (e.g., the N60 at 20–90 ms and N130 at 100–160 ms) were recognized as somatosensory processing of pain, whereas the long latency neural activity (e.g., P260/P300) was probably related to affective and cognitive components (Ploner et al., 1999; Kenntner-Mabiala et al., 2008; Valentini et al., 2013; Hu et al., 2014). By comparing pain intensity ratings along with pain-evoked potentials in pre- and post- MS priming sessions, the influences of MS on pain processing were assessed. The current work would provide insight into a cognitive modulation of pain experience when existential problem occurs.

## Materials and Methods

### Participants

Twenty healthy Chinese male college students participated in the study as paid volunteers. It has been acknowledged that males and females have different pain perception and coping strategies toward pain (Berkley, 1997; Bartley and Fillingim, 2013). To rule out such possible confound of gender, we only recruited male participants in this study. The sample for data analysis included 18 subjects aged between 18 and 27 years old (Mean ± *SD* = 21.3 ± 2.4). Two participants were excluded, with one due to technical failures of EEG recording and the other due to excessive eye blinks or head movements during the experiment. All subjects were right-handed and had no self-reported chronic pain diseases or neurological history. This study was approved by the local ethics committee at the Department of Psychology, Peking University and was carried out in accordance with the approved guidelines. All subjects gave written informed consent in accordance with the Declaration of Helsinki prior to participation.

### Electrical Stimulation

Electrical stimulation was a single 0.5 ms pulse of square waveform and was delivered to the dorsum of the left hand via a pair of foil electrodes (DS7A Digital High Voltage Stimulator [Apparatus], 2009). Sensory and pain tolerance thresholds were determined using the ascending limit method (Niddam et al., 2002; Wang et al., 2014). For each participant, a stimulation of 0.8 mA was applied first and the participant was asked to report whether he could feel the shock and whether he could tolerate a stronger shock. The current intensity was then increased by 0.2 mA each time and the participant was asked to answer the same two questions after each shock. The participant would receive increasing electrical shocks until he answered that he could not tolerate a stronger shock. The sensory threshold was determined as the current intensity with which the participant answered “yes” at the first time to the question “can you feel this shock?” The pain tolerance threshold was defined as the current intensity with which the participant answered “no” at the first time to the question “can you tolerate a stronger shock?”

### Mortality Salience Manipulation

The MS priming procedure was adopted from a previous study (Luo et al., 2014). It consisted of 28 statements and participants had to judge whether he agreed with each of them. These statements were related to death (e.g., “I won’t feel terrible even if I would die lonely.”). As suggested by previous studies (Pyszczynski et al., 1999; Han et al., 2010), death-related thought includes negative emotions such as fear and anxiety and specific mortality processing with engagement of self-awareness. To rule out the influence of general negative emotions, a negative affect (NA) priming procedure was also employed as a control condition, same as in Luo et al. (2014). The NA priming procedure consisted of 28 statements unrelated to death but related to fear or anxiety emotions (e.g., “The coming exam makes me uneasy.”). Each statement appeared on a computer screen for 7 s. When completed all the statements, participants were asked to rate themselves the closeness to death and their NA (i.e., “How close do you feel to death after reading all the sentences and making your judgments?,” “How negative do you feel after reading all the sentences and making your judgments?”). Likert-type scale was used for all ratings where 0 indicated no effect and 10 indicated maximal effect (e.g., “extremely close,” “extremely negative”).

After the priming procedure, the participants performed 40 arithmetic calculations in 5 min. This manipulation was to insert a delay between the priming and painful/non-painful stimulations, during which participants were distracted from the salience of death. According to the TMT, humans employ proximal defenses (distraction or rationalization) and distal defenses (self-esteem or cultural worldview) to defend the death-related thoughts (Pyszczynski et al., 1999; Greenberg et al., 2000). Thus, the distraction period is included aiming at eliciting proximal first and then distal defenses. Importantly, the cognitive modulation of cultural worldviews could be accessible when participants adopt implicit and unconscious “distal” defenses.

### Experimental Procedure

**FIGURE 1:**
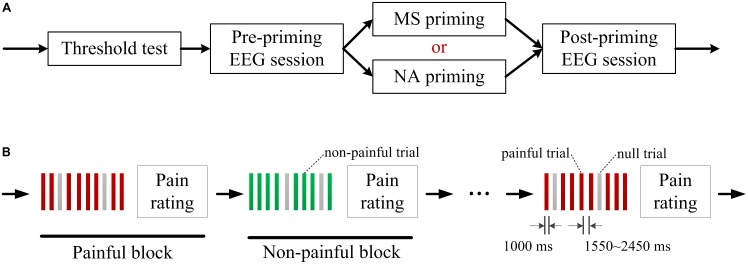
Experimental procedure. **(A)** Each participant received a set of mortality salience (MS) priming on 1 day and a set of negative affect (NA) priming on the other day. A sensory and pain tolerance thresholds test and two electroencephalogram (EEG) sessions were conducted on each day. **(B)** Each EEG session consisted of alternately presented 10 painful blocks and 10 non-painful blocks. Each block consisted of 8 painful/ non-painful electric shocks and 2 randomly assigned null trials. Each trial started with presentation of a square to indicate an electrical shock in 1000 ms and the trial interval varied randomly between 1550∼2450 ms. After each block, participants were asked to rate pain intensity on a 11-point visual analog scale.

Each participant received a set of MS priming on one day and a set of NA priming on the other day. The order of the two priming conditions was counterbalanced across participants. On each day, participants underwent the following tasks successively: sensory and pain tolerance thresholds test, pre-priming electroencephalogram (EEG) session, MS or NA priming task, and post-priming EEG session (**Figure 1A**).

In each EEG session, 80 painful and 80 non-painful stimuli were delivered to the participants. The painful and non-painful stimuli were determined as the current intensities of sensory and pain tolerance thresholds in the threshold test, respectively. The intensities used in present study did not differ between the two priming conditions by paired samples *T*-test (Painful shock: *t*(17) = 0.88, *p* = 0.392; Non-painful shock: *t*(17) = −1.10, *p* = 0.285; see **Table 1**). As illustrated in **Figure 1B**, each EEG session consisted of 10 painful blocks and 10 non-painful blocks, which were alternately presented. Each block consisted of 8 painful/ non-painful electric shocks and two null trials with no shock being delivered. The null trials were randomly assigned to minimize the habituation effect of stimuli with the same intensity. Each trial started with presentation of a square at the center of a computer monitor, as a cue indicating that an electrical shock or a null shock would be delivered in 1000 ms. The interval between two consecutive trials varied randomly between 1550∼2450 ms. After each block, participants were asked to rate pain intensity of the 8 electrical stimulations on a 11-point visual analog scale (0 = no sensation, 1 = feel something but not pain, 4 = slight pain, 8 = strong pain, 10 = worst imaginable pain).

**Table 1:** Current intensities (mA) of electrical shocks used in this study.

	MS priming	NA priming
Painful shock	9.29 ± 3.27	9.61 ± 3.71
Non-painful shock	1.65 ± 0.24	1.57 ± 0.29

### EEG Recording and Data Analysis

Electroencephalogram (EEG) recordings and pre-processing of raw EEG data were similar to our previous study (Wang et al., 2014). EEG data were recorded by 62 Ag-AgCl electrodes in accordance with the extended 10–20 system, with the linked left and right mastoids served as a reference. Two additional electrodes were adopted to record the horizontal (HEOG) and vertical (VEOG) electrooculograms with an aim of monitoring eye movement. The impedance of all electrodes was kept less than 5 kΩ. EEG signal was recorded at a sample rate of 500 Hz and filtered with a band pass of 0.05–100 Hz.

EEG data were processed with SCAN 4.3 software^1^ (v4.3) compatible with the recording product SynAmps Neuroscan, and were further analyzed with MATLAB^2^ (R2014b). During pre-processing, EEG data were first offline filtered (band pass: 0.1–40 Hz, 24dB) and detrended. Second, evoked potentials were extracted with an epoch 200 ms before the onset of an electrical stimulation and lasting for 1000 ms. Third, using MATLAB, we removed the artifact at the stimulus onset caused by the electric stimulator, similar to Zaslansky et al. (1996), and improved the signal at 0∼20 ms around the stimulation by cubic spline interpolation, similar to Christmann et al. (2007). Last, we excluded trials with potentials exceeding ± 50 μV over either HEOG or VEOG electrodes. This Artifact rejection resulted in 47∼58 trials remained for further analysis in each condition. It was not significantly different between conditions. The overall acceptance rate of trials was 66.6%.

Grand averages of EEG were conducted from all electrodes. To overcome the multi-comparison problem, a cluster-level correction method was adopted as characterized pain-evoked potentials in neighboring electrodes being clustered as a region-of-interest. Thus the regions-of-interest were identified as the frontal (FP1, FPz, FP2, F1, Fz, and F2), left frontal-central (FC1, FC3, FC5, C1, C3, and C5), right frontal-central (FC2, FC4, FC6, C2, C4, and C6), and parietal-occipital (P3, Pz, P4, PO3, POz, and PO4) areas. In order to conduct statistical analyses, mean amplitude was calculated within its typical time window for each component, i.e., N60 (50–80 ms), P90 (70–100 ms), N130 (110–140 ms) and P260 (240–320 ms). To test the effects of MS on pain processing, repeated-measures analyses of variances (ANOVAs) were conducted on both ERP amplitudes and subjective pain intensity rating, with Priming (MS vs. NA), Sequence (pre- vs. post-priming), and Stimulus Intensity (painful vs. non-painful). *Post hoc* analyses were further conducted to examine the direct effect of MS on pain-evoked potentials and pain intensity rating. To verify the contralateral feature of somatosensory processing of pain, the Hemisphere (the left vs. right frontal-central area) was considered as a factor into the 4-way ANOVA conducted on ERP components.

In addition, as we hypothesized that MS may reduce pain intensity rating, the reduction effect of each priming was measured by its original and standardized effect size (ES, Cumming, 2014). The original ES of reduced pain intensity was defined as intensity rating recorded in pre-priming session minus that recorded in post-priming session. Confidence interval (CI) reported along with the ES referred to 95% CI. The standardized ES was defined as Cohen’s d:

$$d=(M_{\mathrm{pre}}-M_{\mathrm{post}})/S_{\mathrm{pre}},$$

where *M_pre_* and *M_post_* were the means of pain intensity ratings in pre- and post-priming sessions, and *S_pre_* was the standard deviation of pain intensity rating in pre-priming session.

## Results

### Behavioural Performance

The ratings of MS were 6.3 ± 0.7 after MS priming and were 2.6 ± 0.8 after negative affect (NA) priming. Paired *t*-test confirmed greater amount of death-related thoughts after MS priming compared to NA priming [*t*(1,17) = 5.39, *p* < 0.001]. Meanwhile, the negative feelings induced by priming tasks were not significantly different (MS priming: 3.3 ± 0.6, NA priming 3.8 ± 0.5, *t*(1,17) = −1.04, *p* = 0.313). These results demonstrated a successful manipulation of MS in our sample.

Subjective pain ratings of electrical shocks during EEG recordings were listed in **Table 2**. The ANOVA of Priming, Sequence and Stimulus Intensity on pain ratings revealed that, the main effect of Stimulus Intensity was significant (*F*(1,17) = 1301.93, *p* < 0.001), indicating that painful electrical shocks caused more intensive painful feelings than non-painful electrical shocks. The main effect of Sequence was also significant (*F*(1,17) = 6.90, *p* = 0.018), indicating an habituation effect that participants rated electrical shocks as less painful in the post-priming than in the pre-priming sessions. However, it resulted in an insignificant main effect of Priming (*F*(1,17) = 1.75, *p* = 0.204), indicating that subjective pain ratings did not differ in general between the two priming conditions. The interaction of Priming × Sequence × Stimulus intensity on subjective pain ratings did not reach a significant level (*F*(1,17) = 3.23, *p* = 0.090). Separately, the two-way interaction of Priming × Sequence on pain ratings was marginally significant to painful shocks (*F*(1,17) = 3.86, *p* = 0.066), but insignificant to non-painful ratings (*F*(1,17) = 0.16, *p* > 0.5). Nevertheless, paired *t*-tests revealed that MS decreased pain intensity ratings of painful shocks (pre-priming: 8.48, post-priming: 8.10; *t*(17) = 2.37, *p* = 0.030). In contrast, the intensity ratings in the pre- and post-NA priming sessions did not differ (pre-priming: 8.45, post-priming: 8.42; *t*(17) = 0.39, *p* > 0.5).

**Table 2:** Subjective rating scores of electric shocks.

	MS priming		NA priming	
	Pre-priming	Post-priming	Pre-priming	Post-priming
Painful shock	8.48 ± 0.73	8.10 ± 0.99	8.45 ± 0.86	8.42 ± 0.92
Non-painful shock	1.35 ± 0.40	1.27 ± 0.32	1.42 ± 0.40	1.28 ± 0.35

To further evaluate the reduction effect of each priming on pain intensity, we calculated its original and standardized effect size (ES). The original ES of reduced pain intensity in MS priming was 0.38, with a 95% CI of [0.07, 0.70]. The standardized ES as indicated by the Cohen’s *d* was 0.52, demonstrating a moderate effect (Cumming, 2014). However, for NA priming, the original ES was 0.03 with a 95% CI of [−0.14, 0.21] and the Cohen’s *d* was 0.03. These results indicate that MS priming induced a moderate effect on pain intensity reduction; however, this reduced effect might be constrained by the current experimental settings.

### ERP Results

**FIGURE 2:**
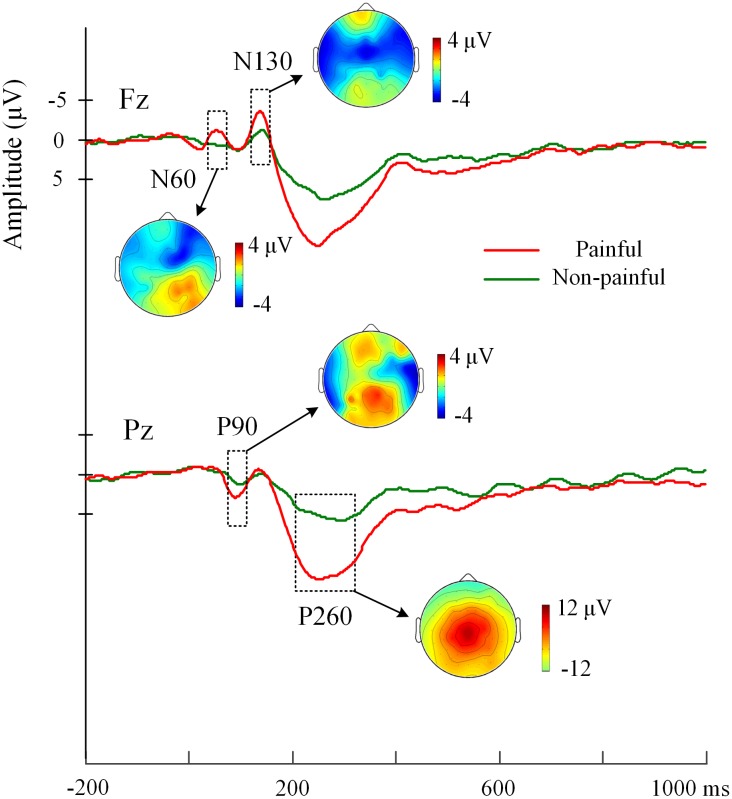
Grand-average EPRs elicited by electrical painful and non-painful stimulations. ERPs at electrodes Fz over frontal region and Pz over parietal region are represented as examples. The pain-evoked potentials are characterized by components, i.e., N60, P90, N130, and P260. The scalp distribution of these ERP components is illustrated by potential topographies.

**Figure 2** illustrated the grand average event-related potentials (ERPs) elicited by electrical painful and non-painful shocks. The pain-evoked potentials were characterized by two successive negative components, i.e., the N60 (50–80 ms) and N130 (110–140 ms) over the frontal/central sites, and a positive component P90 (70–100 ms) over the posterior sites, which were followed by a whole-brain positive component P260 (180–380 ms, peaking at 260 ms). It could be clustered as an early negative complex (N60-P90-N130) and a late positivity (P260).

Analyses of variances of Priming, Sequence and Stimulus intensity on pain-evoked potentials were conducted. Significant main effects of Stimulus Intensity were found, confirming that painful relative to non-painful electrical shocks elicited larger amplitudes at the N60 over the right frontal-central region (*F*(1,17) = 10.45, *p* = 0.005), the P90 over the parietal-occipital region (*F*(1,17) = 22.30, *p* < 0.001), the N130 over the bilateral frontal-central region (Fs(1,17) = 16.12 and 26.54, ps < 0.001), and the P260 over whole brain areas (Fs(1,17) = 39.98 to 100.74, *p*s < 0.001). Moreover, since electrical stimulations were delivered to the left hand, the 4-way ANOVA with Hemisphere as a factor revealed that larger amplitudes were observed over the right than the left frontal-central region at the N60 (*F*(1,17) = 25.53, *p* < 0.001), P90 (*F*(1,17) = 32.34, *p* < 0.001), N130 (*F*(1,17) = 13.00, *p* = 0.002), and P260 components (*F*(1,17) = 5.26, *p* = 0.035).

**FIGURE 3:**
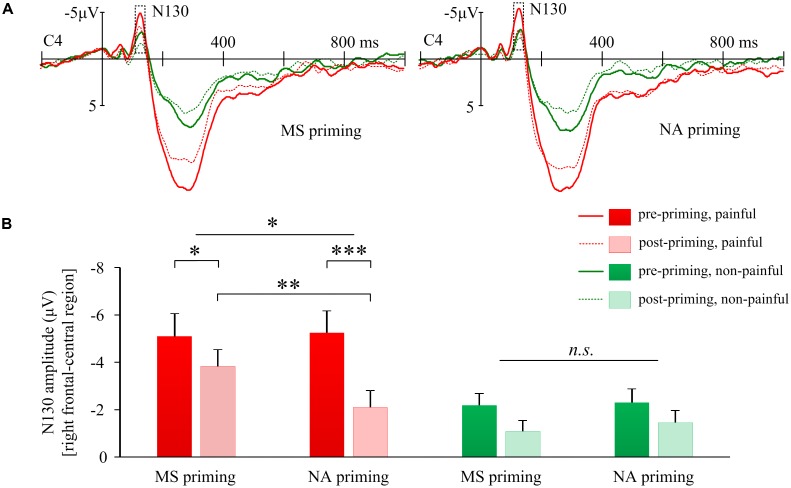
Effects of mortality salience on pain related N130 amplitudes. **(A)** Illustration of grand-average EPRs over C4 elicited by painful and non-painful shocks in mortality salience (MS) priming and negative affect (NA) priming conditions. **(B)** Bars represent mean N130 amplitudes over right frontal-central region in different conditions. Error bars represent standard errors. ^∗^*p* < 0.05, ^∗∗^*p* < 0.01, ^∗∗∗^*p* < 0.001; and n.s., not significant.

We were particularly interested in how MS compared to negative affect influenced pain-evoked potentials. A significant triple interaction of Priming × Sequence × Stimulus Intensity was found on the N130 over the right frontal-central region (*F*(1,17) = 13.66, *p* = 0.002, **Figure 3A**). Separately, for the N130 induced by painful shocks, the interaction of Priming × Sequence was significant (*F*(1,17) = 6.93, *p* = 0.017, **Figure 3B**); whereas for N130 induced by non-painful stimulations, no interaction was found between Priming and Sequence (*F*(1,17) = 0.30, *p* > 0.5). *Post hoc* analyses confirmed that pain-evoked N130 was smaller in post-priming compared to pre-priming sessions, observed in both MS priming (*t*(17) = −2.19, *p* = 0.043) and NA (*t*(17) = −5.59, *p* < 0.001) conditions, which may reflect an habituation effect of painful stimulations. However, pain-evoked N130 after MS priming was larger than that after NA (*t*(17) = 2.90, *p* = 0.010, **Figure 3B**), suggesting that MS may have an opposite effect against habituation.

**FIGURE 4:**
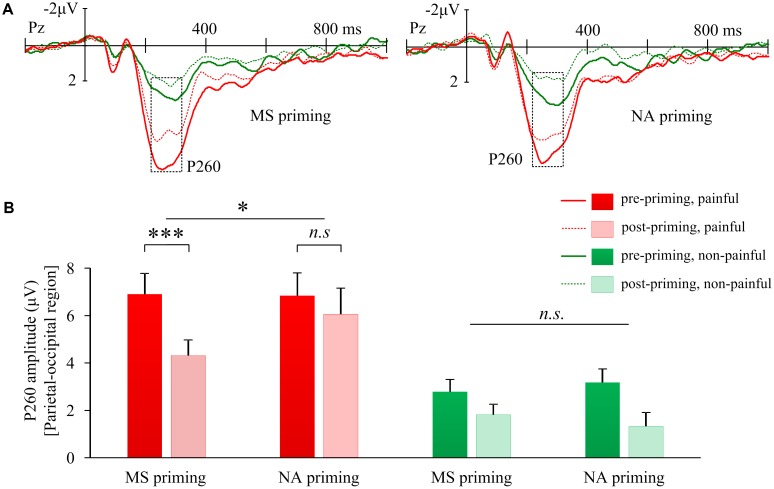
Effects of mortality salience on pain related P260 amplitudes. **(A)** Illustration of grand-average EPRs over Pz elicited by painful and non-painful shocks in mortality salience (MS) priming and negative affect (NA) priming conditions. **(B)** Bars represent mean P260 amplitudes over parietal-occipital region in different conditions. Error bars represent standard errors. ^∗^*p* < 0.05, ^∗∗∗^*p* < 0.001; and n.s., not significant.

The interaction of Priming × Sequence × Stimulus intensity on P260 amplitudes over the parietal-occipital region was also significant (*F*(1,17) = 17.98, *p* = 0.001, **Figure 4A**). Further, the interaction of Priming × Sequence was significant for P260 induced by painful shocks (*F*(1,17) = 4.46, *p* = 0.050, **Figure 4B**); but not for P260 induced by non-painful stimulations (*F*(1,17) = 1.99, *p* = 0.177). *Post hoc* analyses confirmed that compared to pre-priming, pain-evoked P260 was smaller after MS priming (*t*(17) = −4.54, *p* < 0.001). However, NA did not reduce pain-evoked P260 amplitudes (*t*(17) = 1.36, *p* = 0.191). In addition, the 3-way interactions on P260 were insignificant over other regions (frontal: *F*(1,17) = 0.28, *p* = 0.603; left frontal-central: *F*(1,17) = 0.62, *p* = 0.441; right frontal-central: *F*(1,17) = 1.58, *p* = 0.226). These results suggest that the decreased P260 over the parietal-occipital region by MS may reflect a cognitive modulation on pain. Moreover, the change of parietal-occipital P260 amplitude after MS priming (ERP amplitude in post-priming condition minus that in pre-priming condition) was positively correlated with the change of right frontal-central N130 amplitude (*r* = 0.48, *p* = 0.044), indicating that the two components co-vary with each other when subjecting to the modulation of MS.

## Discussion

Pain-evoked potentials were identified in our study as an early negative complex (N60-P90-N130) and a late positivity (P260), which was consistent with previous findings (Babiloni et al., 2001; Christmann et al., 2007; Wang et al., 2014). These components exhibited a contralateral characteristic of sensory processing. Importantly, compared to the NA, the MS priming resulted in enhanced pain-evoked N130 and decreased P260 components. In parallel with the pain-evoked P260, subjective pain intensity ratings might also be changed by MS. The findings indicate that reminders of mortality affect both early sensory and late cognitive neural responses to nociceptive stimuli, which may reflect a cognitive modulation of pain.

The influence of MS on subjective rating of pain intensity is probably sensitive to specific mental state or cultural context. As Valentini et al. (2014, 2015) reported an increased effect of MS on pain rating in Westerners, we found an unchanged or possibly reduced effect in Chinese. In combination with the TMT (Greenberg et al., 2000), the inconsistent findings here imply that individuals in different culture may employ diverse cultural worldviews to defend the existence problem. For example, compared to western context, people from East Asian culture prefer low arousal emotions. Such cultural preferences for lower arousal emotions may serve to decrease pain perception, whereas cultural norms favoring catastrophizing may serve to increase pain perception (Anderson and Losin, 2017). Moreover, the long-term orientation/ Confucian (Hofstede, 2001) and religious coping (Anderson and Losin, 2017) in Chinese culture might also be associated with decreased pain perception. Apart from cultural context, subliminal manipulation of MS could further change the effect on pain rating. When death-related thoughts were fast and automatically accessed by pictures, no change in pain ratings was found (Valentini et al., 2017). Nevertheless, it is noteworthy that these studies adopted different paradigms to track pain-evoked potentials and this might also contributed to the discrepancy.

In our study, the N130 amplitudes elicited by painful electrical shocks decreased in the post-NA priming sessions compared to pre-NA priming sessions. This replicated previous findings of a habituation effect of pain-evoked potentials when repetitive painful stimulations were applied (Miltner et al., 1987; Bingel et al., 2007; Valentini et al., 2014). Interestingly, we found that the habituation effect on the N130 amplitudes over the right frontal-central region was, to some extent, eliminated when participants were primed with MS. The N130 component is probably related to somatosensory processing of pain arising from the contralateral SI/SII (Ploner et al., 1999), which could be much affected by consciousness (Wang et al., 2003). We speculate that MS may induce anxiety about the body existence, which may further give rise to special attention to painful stimulus (Mogg et al., 1992). Our findings suggest that reminders of mortality enhance early somatosensory activities to painful shocks.

The pain-evoked P260 amplitudes over the parietal-occipital region decreased compared to the baseline, after MS priming but not after NA priming. A habituation effect of repetitive nociceptive input was considered to contribute to the decrease of P260 component for two reasons. First, the P260 in the present study exhibited a contralateral feature of stimulation site over the frontal-central region, indicating that the P260 was involved in somatosensory processing of pain, a component that could be easily subjected to the habituation effect. Second, similar with the P260, the pain-evoked N2-P2 components elicited by other types of nociceptive stimuli have been found to be reduced by stimulus repetition (e.g., contact-heat, Greffrath et al., 2007; laser, Iannetti et al., 2008). Thus, the results could be explained as MS priming, relative to NA priming, increased the habituation effect of pain-evoked P260 component. We speculate that this might be due to a cognitive modulation of MS on pain processing.

The P260 has been recognized as a component related to top-down cognitive modulation of pain processing (Lorenz and Garcia-Larrea, 2003; Kenntner-Mabiala et al., 2008; Valentini et al., 2013). For example, pain-evoked P260 was enhanced by an intentional focus on the stimulus intensity (Kenntner-Mabiala et al., 2008). It was also reported that a pain-evoked P2a component (260–360 ms) decreased when high hypnotically suggestible individuals were provided with hypnotic suggestions of down-regulating pain unpleasantness (Valentini et al., 2013). Thus, decreased P260 in our study might reflect an intentional down-regulation of the pain intensity and unpleasantness, which resulted in an increase of habituation effect. It is likely that when being reminded of MS, Chinese participants may embrace a long-term orientation/ Confucian cultural worldview and then may reappraise of the meaning of pain as less threatening, since previous studies revealed that P260 and late positive potential could be involved in threat processing (Bar-Haim et al., 2005; Miltner et al., 2005; Bublatzky and Schupp, 2011). As a consequence, the repetitive nociceptive inputs are perceived as more endurable, which may lead to the observed increase of habituation over the P260. Hence, our finding adds new evidence into the role of top-down cognitive modulation in determining the habituation of pain neural responses (Valentini et al., 2011).

It is intriguing that the N130 and P260 components carry opposite effects of MS on pain processing. The increased N130 might be explained as individuals paid special attention to the nociceptive stimulus at an early stage when encountered with death-related thoughts; whereas the decreased P260 might be explained as they down-regulated the significance of the threatening input at a later stage. Finally, individuals reported subjective pain intensity with a decreased trend. Moreover, it is claimed in previous study that pain-evoked potentials are mainly determined by the saliency but not the perception of pain *per se* (Iannetti et al., 2008). Thus, the plausible contradicting observations of behavioral report and brain responses can be reconciled. In addition, another argument is that the N130 and P260 components may be an overlap temporally. Indeed, a significant correlation was observed between the change of the N130 and that of P260, suggesting that the MS could have shifted the scalp signal toward the negative direction, which lead to both an increase of N130 amplitude and a decrease of P260 amplitude. However, we believe that the N130 and P260 are though interconnected but two different components, as they are found to subject to the MS effect in different regions (right frontal-central N130 vs. parietal-occipital P260) and serve for different functions.

There are some limitations in the current study. First, our findings were merely dependent on a male sample. It was reported that females were more willing to report pain (Robinson et al., 2001) and to share painful experience with others to get social support than males (Bartley and Fillingim, 2013). As cultural/social norms favoring catastrophizing lead to increased pain intensity, diverse influences of MS on pain perception might be expected between genders. Second, the intensity of the non-painful stimuli used in the present study were much lower than that of painful stimuli. It could be a potential confound when detecting the 3-way interaction, as the signal-to-noise ratio was usually lower for lower brain responses elicited by low-intensity stimuli. In future investigation, it would be better to add another control condition by employing intensity-matched non-painful stimuli, such as auditory stimulation that has been adopted in other studies (Valentini et al., 2014). Third, there was no direct evidence to show that different culture-specific worldviews were adopted to cope with pain when people in different cultures encountered death-related thoughts. This leaves an interesting question about the relationship among MS, cultural worldview and pain processing for future cross-cultural studies. Last, another limitation is its relatively small sample size. This may reduce the power of the study and thus might be responsible for the marginal significant effect in behavioral results.

The current work extends the understanding of the biopsychosocial factors of pain (Gatchel et al., 2007). It has been documented that pain processing can be affected by both bottom-up and top-down processes, such as habituation (Ernst et al., 1986), attention (Kakigi et al., 2005), self-regulation (Woo et al., 2015) and anticipation (Yu et al., 2014). It was also found that cognitive regulation, such as mindfulness meditation, was effective for chronic pain intervention (Morone et al., 2008; Zeidan et al., 2011). Moreover, sociocultural context, such as independent/interdependent self-construals, affects the neural activity of pain perception (Wang et al., 2014). In this study, we demonstrated that both pain intensity and pain-related brain activities are manipulated when death-related thoughts are accessible.

The current work also complements the impacts of MS on perception, cognition and neural activities. Previous ERP studies reported modulations of an early frontal activity and a late parietal activity by perceived death-related vs. death-unrelated words (Klackl et al., 2013; Liu et al., 2013). Moreover, MS reduced the frontal activities to racial ingroup faces (Henry et al., 2010) and the anterior cingulate activity in responses to empathy for pain in others (Luo et al., 2014). By showing the modulation on pain evoked potentials, our study extends the influences of death-related thoughts on mental processing from high-level cognition to low-level pain perception.

In summary, MS modulates pain evoked potentials, i.e., the N130 and P260, which are, respectively, associated with early sensory processing and late cognitive modulation of physical pain. Our findings indicate a down-regulation effect of MS on physical pain in a Chinese male sample, which provides insight into a culture-sensitive perspective on how pain experience would be modulated when one is faced with existential problems.

## Data Availability Statement

Datasets are available on request. The raw data and generated data during analyses supporting the conclusions of this manuscript will be made available by the authors, without undue reservation, to any qualified researcher.

## Author Contributions

CW and JT designed the study, collected and analyzed the data. CW wrote the manuscript. All listed authors contributed to manuscript revision and have approved the final manuscript.

## Conflict of Interest Statement

The authors declare that the research was conducted in the absence of any commercial or financial relationships that could be construed as a potential conflict of interest.

## Footnotes

1 http://www.neuroscan.com/

2 https://www.mathworks.com/
